# Noninvasive Cardiovascular Imaging in Preclinical Research: Do We Get New Biomarkers Usable in Humans?∗

**DOI:** 10.1016/j.jacbts.2023.02.009

**Published:** 2023-07-24

**Authors:** Frank Kober

**Affiliations:** Aix-Marseille Univ, Centre National de la Recherche Scientifique (CNRS), Centre de Résonance Magnétique Biologique et Médicale (CRMBM), Marseille, France

**Keywords:** atherosclerosis, imaging, immunology, inflammation, MI

**Figure:**
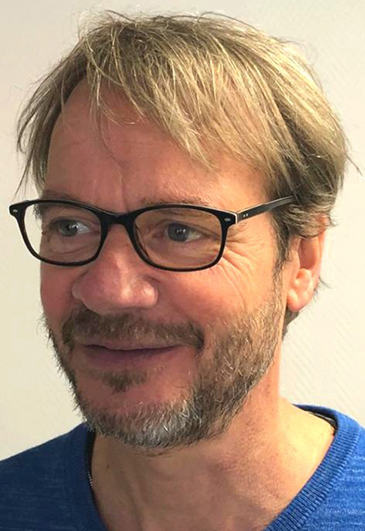

Localized and noninvasive information on inflammatory processes is important for understanding the pathophysiology of many diseases, especially at early stages. In heart disease, it may allow better targeting of therapies reducing secondary tissue damage, especially after myocardial infarction (MI). In atherosclerosis, knowing the level of inflammation is a potential key for predicting the evolution of plaque and may therefore help guide therapy. Among the many preclinical imaging techniques that were used in the past for imaging inflammation, both magnetic resonance imaging (MRI) and positron emission tomography (PET) have a strong translational potential, because they can do highly specific imaging deep inside the body.

The work by Maier et al^1^ in this issue of *JACC: Basic to Translational Science* contributes to evaluating several potential biomarkers of the immune system’s responses after MI. Using several imaging techniques in combination, they also provide new data relevant for the understanding of inflammation processes after MI in the myocardium, in bone marrow, and in atheromas. Although this is an entirely preclinical study on mice, ^19^F MRI and PET with 4 tracers with different specificities were used, all of which are technically feasible on the human scale. Here, the techniques yielded highly significant results on the dynamics of myeloid cell influx and monocyte recruitment after MI, and the findings were validated using flow cytometry.

The investigators compared 2 MI mouse models, one with permanent and the other with a (clinically more relevant) transient coronary artery occlusion. Similar to a clinical session for detecting myocardial viability, both ^18^F-fluorodeoxyglucose PET and late gadolinium enhancement MRI allowed them to demonstrate the short-term differences between the 2 occlusion models: transient occlusions produce smaller infarcts. More important, it allowed them to pursue their experiments from a well-characterized starting point.

The main focus of their study was to give insight into immune processes after MI, and to this end, they first used ^19^F MRI with a specific lipoprotein-based nanotracer of immune cells injected via the tail vein. The higher ^19^F MRI signal obtained in infarct regions after both coronary occlusion procedures suggests myeloid cell influx into these regions. This was in line with higher CD11b^+^ cell burden (visible on the ^89^Zr nanobody positron emission tomographic images targeting CD11b^+^ cells) and higher inflammatory monocyte recruitment visible in the ^64^Cu positron emission tomographic images targeting the CCR2 receptor. Again, the signals showed variations with the type of infarction, indicating differences in the severity of the immune response. All additional assays the investigators performed to validate their in vivo imaging results confirmed these findings. On a quantitative level, they correlated the myeloid cell burden obtained on flow cytometry in both models with global cardiac function from cine MRI and found a strong inverse correlation, underlining the direct relationship of the inflammation following MI with cardiac function.

Because immune cells delivered to the heart are produced in the bone marrow and in the spleen, they also analyzed different positron emission tomographic signals in the spinal and splenic regions, comparing the 2 occlusion models. They found significantly lower myeloid cell signal and higher metabolic activity after infarction, but the response was similar between the permanent and the transient occlusion model. CCR2 expression did not increase in the bone marrow, which the investigators attribute to their likely immediate departure from the site. In the spleen, they could also use the ^19^F MRI signals, which were lower than in control animals only in the permanent occlusion situation but not after transient occlusion. CD11b^+^ PET, however, showed lower signal in both models, and the investigators open doors toward further research to elucidate this apparent difference between the 2 detection methods.

Finally, the investigators also assessed the impact of MI on atherosclerotic plaque development in the 2 infarction models in vivo. Because an atherosclerosis-accelerating effect of the inflammation following MI was shown in an earlier study,^2^ they aimed to investigate whether revascularized infarction has a similar impact. They used ApoE^−/−^ mice fed with a Western diet for 6 weeks as an efficient model of atherosclerosis. In this part of the study, MRI was used only at the time point 2 days after the infarct to determine cardiac function and infarct extent. Four weeks after infarction, they used flow cytometry to count inflammatory monocytes in spleen, femur bone marrow, and aorta. Globally, both monocyte counts and plaque burden evaluated on histology in the aortic root were higher in both infarction situations compared with control mice that had not undergone coronary ligation, showing that atherosclerosis significantly accelerates after ligation, be it transient or permanent.

Among the in vivo imaging techniques used, perfluorocarbon ^19^F MRI appears to be a particularly attractive candidate for translation to the clinic because of the absence of radiotracers. Unlike PET, imaging inflammation noninvasively has been a challenge with conventional MRI, because contrasts based on T1, T2, or T2∗ relaxation times are submitted to other contributors in altered tissue. They are therefore submitted to radiologic interpretation and experience. Late gadolinium enhancement MRI features high spatial resolution but is used mainly to delineate the extent of the infarcted area, not for a deeper analysis of the zone itself. ^19^F MRI offers the possibility to depict specific processes without “background” signal thanks to targeting fluorinated tracer molecules. Perfluorocarbons are considered biologically inert and seem well suited for explorations in humans. From a technical point of view, ^19^F MRI detection requires specific radiofrequency antennae currently not supplied with clinical MRI systems. A recent in vivo study in swine has shown that clinical 3.0-T scanners are able to depict the perfluorocarbon fluorine signal in the infarcted myocardium within 20 minutes,^3^ which can be compared with the mouse imaging at 9.4-T in this study, in which the investigators obtained 3-dimensional ^19^F images from the mouse myocardium in vivo within about 30 minutes. Technically, cardiac ^19^F perfluorocarbon MRI in humans therefore seems feasible. Despite these promising findings and facts, past human ^19^F MRI applications have remained limited to imaging inhaled fluorinated gas in the lung.

Conventional ^18^F-fluorodeoxyglucose PET has a longer history of clinical use in imaging inflammation of various types, including cardiac disease in humans (for a recent example see Tessier et al^4^) and atherosclerosis.^5^ Inflammation-specific positron emission tomographic tracers are also being used in clinical research studies. But the specific tracers for CD11b^+^ cell burden and CCR2 receptor in particular bear translational potential, and the results obtained in this study underline their usefulness in cardiovascular research. They will help guide future protocols in clinical research but also in further fundamental preclinical work.

From a purely preclinical and fundamental point of view, Maier et al^1^ provide new insights into the role of inflammation in myocardial tissue remodeling after infarction on one hand and in the known risk for another episode of infarction on the other hand. They extended their known earlier findings of accelerated atherosclerosis after infarction to a more realistic infarction model (ischemia-reperfusion) and showed that the earlier findings apply just as well. To draw their important conclusions, they used an impressive arsenal of noninvasive preclinical imaging approaches together with cytometry and histology for subsequent validation. The results may, as such, stimulate the development of therapeutic approaches with inflammation as a narrow target. But the preclinical methodology itself, which they proved to work, can be seen as a result in form of a powerful tool to help validate new therapeutic approaches. Moreover, the noninvasive and repeatable imaging methods here appear to be potential future clinical biomarkers that may contribute to better follow-up of patients using imaging with higher specificity.

## Funding Support and Author Disclosures

This work received support from Centre National de la Recherche Scientifique (CNRS UMR 7339) and France Life Imaging Network (ANR-11-INBS-0006). The author has reported that he has no relationships relevant to the contents of this paper to disclose.
